# Phenotyping and genotyping are both essential to identify and classify a probiotic microorganism

**DOI:** 10.3402/mehd.v24i0.20105

**Published:** 2013-03-11

**Authors:** Gianfranco Donelli, Claudia Vuotto, Paola Mastromarino

**Affiliations:** 1 Microbial Biofilm Laboratory, Fondazione Santa Lucia IRCCS, Rome, Italy; 2 Department of Public Health and Infectious Diseases, Section of Microbiology, Sapienza University of Rome, Italy

**Keywords:** probiotics, microorganisms, taxonomy, identification, genotyping, phenotyping

## Abstract

The use of probiotic products, especially for humans, requires an unequivocal taxonomical definition of their microbial content, in order to assign the probiotic effects to well identified and characterized microbial strains. In the absence of this, the labeling of some marketed probiotics may be misleading, both in terms of microbiological contents and possible beneficial effects. Currently, the ‘polyphasic taxonomy’ based on the integration of phenotypic and genotypic data seems to be the most appropriate approach. In fact, even if phenotypic characters often overlap among genetically different species, the molecular methods alone are frequently not able to establish distinct boundaries among phylogenetically related species. Thus, a valid scheme for the identification of a probiotic strain should be currently based on its morphological, physiological, and biochemical features as well as on aspects of its genetic profile. It is important that the identity of specific probiotic strains appearing on the product label is the result of a carefully selected combination of suitable phenotypic and genotypic analytical methods. Only adoption of such a policy could give the right emphasis to the significance of strain-specificity and thus provide health authorities with accurate tools to better evaluate the health benefits claimed by each probiotic-based product. The most common phenotypic and genotypic methods are briefly reviewed here with the aim of highlighting the suitable techniques which can be used to differentiate among microorganisms of probiotic interest, particularly those claiming beneficial health effects for humans.

The phenotypic characterization of microbial strains used as probiotics has, for many years, represented the only identification approach, although this methodology always leaves uncertainties and difficulty of interpretation. In fact, a number of papers have described inaccuracies in phenotype-based speciation of microbial strains contained within a probiotic product. Yeung and colleagues reported in 2002 (1) some typing discrepancies occurring in *Lactobacillus acidophilus* and *Lactobacillus casei* groups by using phenotype-based techniques.

Lactobacilli and bifidobacteria have traditionally been identified on the basis of cell morphology, analysis of fermentation products and related enzymatic activities, and by their ability to utilize different carbohydrate substrates. The identification and classification of lactic acid bacteria by using these phenotypic approaches have been reviewed and their limited reproducibility has been discussed (2–4). Particularly, carbohydrate fermentation analysis was considered not sensitive enough to identify species within the *L. acidophilus* group. Fatty acid methyl ester (FAME) analysis was also found to be inaccurate and not recommendable for the identification of lactobacilli.

Currently available molecular techniques, fortunately, provide an important contribution to definitely identifying and classifying microorganisms on the basis of their genotypic characteristics.

The use of probiotic products, especially for humans, requires an unequivocal taxonomical definition of their microbial content, in order to assign the probiotic effects to well-identified and characterized microbial strains. Without this, the labeling of some marketed probiotics may be misleading, both in terms of microbiological content and possible beneficial effects. In addition, proper characterization is important to maintain consumer confidence. Product labels often list invalid names of organisms or misidentify the species the product contains, leading to consumer confusion (5, 6).

Currently, the ‘polyphasic taxonomy’ approach, first proposed by Rita Colwell (7) for the genus *Vibrio* and based on the integration of phenotypic and genotypic data, seems to be the most appropriate one. In fact, even if phenotypic characters often overlap among genetically different species, the molecular methods alone are frequently not able to establish distinct boundaries among phylogenetically related species. Thus, an updated scheme for the identification of a probiotic strain should be currently based on its morphological, physiological, and biochemical features as well as on the investigation of its genetic profile through the following methodologies:

1. DNA–DNA hybridization technique;
2. Amplified ribosomal DNA restriction analysis (ARDRA); and
3. 16S and 23S rDNA sequencing.

Since previous studies have suggested that most probiotic activities are strain-specific (8–11), the microbial identification at the strain level is mandatory, as indicated by FAO/WHO guidelines for probiotic foods (http://www.fao.org/es/ESN/food/foodandfood_probio_en.stm).

The DNA-based techniques most commonly used for strain-specific typing of probiotic microorganisms are:

1. Pulse field gel electrophoresis (PFGE);
2. Random amplified polymorphic DNA-PCR (RAPD-PCR);
3. Amplified fragment length polymorphism (AFLP); and
4. Ribotyping.

Among the protein-based methods, sodium dodecyl sulfate polyacrylamide gel electrophoresis (SDS-PAGE) is the most commonly applied even if literature data indicate that PFGE is more effective in discriminating between probiotic strains because of its higher sensitivity and accuracy (12).

However, considering the continual evolution of molecular methodologies in this field, the list of techniques mentioned above is expected to be integrated in the near future.

All the techniques used in identification and genetic characterization of microorganisms contained in a probiotic product have to be notified to the control authorities before marketing and, in light of the strain specificity of the probiotic effect, the microbial content of the product at strain level must be maintained unchanged. Further, as suggested by FAO/WHO 2001 document (ftp.fao.org/es/ESN/food/foodandfood_probio_en.stm), microbial taxonomy has to refer to the International Code of Nomenclature recognized by the International Union of Microbiological Societies (IUMS) and strains must be deposited in a renowned international culture collection so as to be available on request for scientific purposes and quality controls.

In this review, the most common and relevant typing techniques will be described by discussing their advantages and limits and evaluating their applicability, reproducibility, and ease of use.

## Phenotypic methods

Phenotypic characterization of probiotic strains is based on data supplied by all the typing methods not based on DNA or RNA, including chemotaxonomic methods that are able to give information on chemical constituents of microbial cells. Thus, the classical phenotypic tests are an important source of data for a preliminary description of taxa, from species up to genus and family. In fact, in many cases the set of all the morphological, physiological, and biochemical features of a strain allows a recognition of taxa. However, these phenotypic characteristics in specific microbial groups, such as lactobacilli and bifidobacteria, are not enough to completely describe or differentiate taxa and must be accompanied by a genotypic analysis (13, 14).

The morphological investigation of a microorganism both by light and electron microscopy provides information on cell shape, flagella and inclusion bodies while color, dimension, and form of its colonies are detected macroscopically on a suitable agar plate. Physiological data useful for classification purposes include growth temperature, pH value, salt concentration and oxygen requirement whereas biochemical features of interest include enzymatic activity, gas production and compound metabolism (15, 16).

According to the most common protocols, carbohydrate fermentation analysis is carried out using API 50 CH (Biomerieux, Italia SpA), a research strip that enables the study of the metabolism of 49 carbohydrates and is able to identify *Lactobacillus* species within 48 hours. However, some epidemiological studies have reported shortcomings in the use of this methodology due to the possibility of identifying lactobacilli belonging to different species as the same microorganism (17). Thus, fermentative profile seems to be an inaccurate method for identification and classification of *Lactobacillus* species which therefore needs to be complemented with a genotypic analysis (18).

FAME analysis (19) has been successful since fatty acids are the major constituents of lipids and lipopolysaccharides in microbial cells and have been extensively used for taxonomic purposes. In fact, more than 300 different chemical structures of fatty acids have been identified and their variability in chain length, double-bond position, and substituent groups has been very useful for the characterization of bacterial taxa (20). This is a cheap and rapid method with a high degree of automation that was recently used to investigate the diversity of 94 *L. reuteri* isolates, arranging them into several clusters (21).

## Genotypic methods

The application of molecular biology techniques has greatly improved our ability in bacterial identification and classification, particularly by genotypic methods directed toward DNA or RNA molecules.

The currently available molecular-based typing methods are mainly founded on restriction analysis of the bacterial DNA, polymerase chain reaction (PCR) amplification of specific genetic targets and identification of DNA sequence polymorphisms. Taking into account our focus on lactobacilli and bifidobacteria, we will review the only techniques able to identify and classify these species as well as those needed to define the strain specificity.

### Species-specific typing

#### DNA–DNA hybridization technique

The DNA–DNA hybridization and the decrease in thermal stability of the hybrid are used to differentiate species, since percent DNA–DNA hybridization is an indirect parameter of the sequence similarity between two entire genomes (22).

None of the most used techniques, such as the hydroxyapatite method (23), the optical renaturation rates method (24), or the S1 nuclease method (25), provide a DNA–DNA hybridization value that can be transformed into a percentage of whole-genome sequence similarity. In fact, by for instance using the S1 nuclease method, the percentage of similarity is calculated by dividing the counts/min of a heterologous nuclease S1-resistant DNA by the counts/min of the homologous S1-resistant DNA and multiplying the resulting number by 100, while the difference between the denaturation temperature of the homologous reaction mixture and that of the heterologous one is indicative of the thermal stability of DNA hybrid.

All the classical techniques need large amounts of DNA and are quite time-consuming. Thus, faster methods requiring less DNA have been described (26, 27). However, DNA–DNA hybridizations are often carried out under conditions not necessarily optimal or stringent for all bacterial DNAs as critically reviewed by Johnson in 1991 (28).

In spite of the above mentioned limitations, the DNA–DNA hybridization technique has been often used, alone or in combination with other phenotypic and genotypic methods, for the identification of bifidobacteria in probiotic dairy products (29) and of Lactobacillus species in various mild yoghurts (30), saliva (31), and supragingival plaque samples (32).

#### Amplified ribosomal DNA restriction analysis

First described in 1992 by Vaneechoutte (33), ARDRA involves an enzymatic amplification using primers directed at the conserved regions in the ends of the small 16S ribosomal bacterial subunit, followed by digestion using tetracutter restriction enzymes. Patterns obtained from several restriction enzymes can be compared with those obtained from reference strains, thus recognizing the strain at species level.

ARDRA has been shown to be the most suitable technique for a rapid discrimination among different species of propionibacteria used as probiotics, being able to classify also the 15% of the 34 studied strains showing atypical profiles when assayed with phenotypic tests of carbohydrate fermentation and peptidoglycan hydrolase activity (34). Moreover, this technique has been successfully applied to the identification of *Lactobacillus* spp. contained in starter and probiotic cultures (35) as well as of lactobacilli, streptococci, and bifidobacteria in commercial fermented milks with bifidobacteria (36).

ARDRA has been recently suggested as the method of choice for distinguishing between *B. longum* ssp. *longum* and *B. longum* ssp. *infantis* in a study on 28 fecal samples of *B. longum* isolated from breast-fed healthy infants (n. 25) and healthy adults (n. 3) and compared with two reference strains (37).

#### 16S and 23S rDNA sequencing

The traditional detection, identification, and classification of lactobacilli based on morphological, physiological and biochemical tests have greatly improved following the introduction of 16S and 23S rDNA sequencing (38–41). In fact, some regions of the 16S rRNA molecule and, to a lesser extent, of 23S rRNAs are conserved throughout all bacterial species and their alignment allows the comparison of the remaining regions (V1 to V9) which are variable with respect to nucleotide base sequence and are sources for species-specific probes or PCR primers to be used for the identification of bacterial species (42–44). Even if much more remains to be done for the species classification of bifidobacteria (45), this molecular approach allows a reliable identification of *Lactobacillus* species (46–49).

### Strain-specific typing

Identification at strain level is necessary since the positive effects that probiotics are able to exercise on the human health cannot be attributed to a genus or species, because the claimed beneficial properties are strain-dependent. Thus, it has become mandatory to exploit all the available molecular methods to reach an unequivocal identification of the probiotic strain.

#### Pulse field gel electrophoresis

First developed by David Schwartz in 1984 (50), this method has been proposed for separating DNA molecules larger than 10 kb. Indeed, unlike the standard method, DNA molecules are subjected to a periodical changing of the electric field polarity that causes the relaxing of DNA molecules in the gel upon the removal of the first field and their elongation to align with the following field.

According to the standardized interpretation system of PFGE patterns suggested by Tenover group in 1995 (51), bacterial isolates yielding the same PFGE pattern can be considered as belonging to the same strain. Isolates differing: 1) by one to three bands are to be considered closely related; 2) by four to six bands possibly related; and 3) by six or more bands unrelated. These criteria are applicable to small, local studies in which genetic variability is presumed to be limited.

Even if PFGE is commonly considered the ‘gold standard’ of genotyping methods, being able to provide a clear identification at the strain level, this approach is not very suitable for routine use, mainly because the procedure needs two to three days to complete.

The usefulness of PFGE to identify different lactobacilli strains in probiotic foods for humans (52) as well as its ability to distinguish 21 different profiles among 39 *L. paracasei* isolates, underlying their high diversity at the strain level (53) was demonstrated.

In a recent study carried out to assess the colonization of vaginal epithelium by an orally administered mixture of *L. fermentum* 57A, *L. plantarum* 57B and *L. gasseri* 57C, PFGE has been used for the identification at the strain level of the different lactobacilli (54).

#### Random amplified polymorphic DNA assay

RAPD is based on the use of short (9–10 bases in length) random sequence primers, which hybridize to chromosomal DNA sequences at low annealing temperatures to be used to start the amplification of regions of bacterial genome. If two primers anneal within a few kilobases of each other and are properly oriented, the result will be a PCR product having a molecular length equal to the distance between the primers. The number and location of these random primer sites is variable from strain to strain of a bacterial species. Thus, visualizing the amplification products by agarose gel electrophoresis, it is possible to obtain a pattern of bands typical of the specific bacterial strain (55, 56).

Numerous publications report on molecular PCR typing performed by RAPD analysis to discriminate both at genus- and strain-level among different probiotic lactobacilli (57–59) and bifidobacteria (36, 60).

#### Amplified fragment length polymorphism

AFLP is a highly sensitive method for detecting polymorphisms in DNA based on the selective amplification of DNA fragments that are obtained by digestion with restriction enzymes (61). Compared to other analytical molecular techniques such as (RAPD)-PCR, AFLP not only has a better reproducibility, a higher resolution and a greater sensitivity at the genome level, but it also exhibits the ability to amplify between 50 and 100 fragments at the same time, so becoming one of the most frequently applied techniques for taxonomical studies of bacteria (62). Recently AFLP has been also used for the strain-specific identification of probiotic strains of lactobacilli (63) and bifidobacteria (64).

#### Ribotyping

Ribotyping is a molecular technique for the identification and classification of bacterial species/strains based upon differences in their rRNA. DNA, after extraction from a colony of bacteria and its restriction into fragments of different lengths by a specific restriction enzyme, is made to run in an agarose gel by electrophoresis. Then, DNA fragments, separated by size, are transferred to a nylon membrane and incubated with a specific enzyme-linked DNA probe (a DNA fragment that hybridizes to the genes coding for 16S and 23s rRNA). After washing, an enzyme substrate, able to produce a fluorescent or chromogenic signal and to visualize the DNA fragments, is added to the nylon membrane and the observed pattern is used for strain identification and classification.

Ribotyping has been applied in the last two decades to differentiate lactobacilli (65–67) while the most recent taxonomic studies have been focused on the probiotic strain *L. rhamnosus* 35 (68) and on the typing of *Lactobacillus* spp. occurring in dental caries (69).

#### Denaturing gradient gel electrophoresis

Denaturing gradient gel electrophoresis (DGGE) is a molecular fingerprinting technique that allows the separation of DNA fragments up to 500 bp in length by using a denaturing gradient gel. Separation of PCR amplified fragments with a similar length is obtained by chemical denaturation resulting in characteristic banding patterns from PCR product mixtures. Individual bands, separated by DGGE, can also be identified by direct cloning and sequencing, or by hybridization with group- or genus-specific DNA probes. This technique is widely used in strain-specific identification of lactobacilli and has been demonstrated to be a winning molecular approach in a large study on the occurrence of *Lactobacillus* species in the gastrointestinal microflora of mice (70). Its usefulness has been also reported in recent studies on the intestinal persistence of orally administered human probiotic *Lactobacillus* sp. strains in healthy adults (71).

However, the high degree of genome conservation observed between some probiotic strains requires more complex and sophisticated analyses to differentiate among strains. As an example, several strains of *B. animalis* subsp. *lactis*, widely used in food products and dietary supplements, cannot be differentiated by a variety of conventional phenotypic and nucleic acid-based techniques, including PFGE and RAPD-PCR (72). Sequencing and comparison of two *B. animalis* subsp. *lactis* genomes (DSMZ 10140 and Bl-04) confirmed the high level of sequence similarity and identified only 47 single-nucleotide polymorphisms (SNPs) and four insertions and/or deletions (INDELs) between them (73). A sequence-based typing method targeting these loci demonstrated that a combination of a reduced number of genetic loci could permit greater discrimination between strains of *B. animalis* subsp. *lactis* than previously attempted methods (72).

## Protein-based methods

Typing methods focusing on the protein profile of bacterial strains have been reported in the literature to differentiate among isolates belonging to diverse species even if these methods have often been used in combination with nucleic acids-based molecular techniques (74).

Such a polyphasic approach has been applied to the identification of 98 strains belonging to nine species of the *L. acidophilus* rRNA-group. Also in this case, in addition to the use of SDS-PAGE of cellular proteins, the molecular analysis needed to be integrated with RAPD-PCR and AFLP with fluorescent primers to achieve a better discrimination between some strains of *L. gasseri* and *L. johnsonii* strains and of *L. amylovorus* and *L. gallinarum* (58).

The use of SDS-PAGE analysis alone to differentiate among three *L. rhamnosus* strains has been also recently reported (75).

## Concluding remarks

All the data obtained on the biological nature of a bacterial strain have important value, particularly in the case of probiotic microorganisms claiming beneficial health effects for humans. In fact, phenotypic and genotypic data as well as the protein profile can significantly contribute to definitively characterize any probiotic both at species and strain level. ‘Polyphasic taxonomy’ is based on the integration of morphological, physiological, and biochemical data together with the molecular ones. The list of the most common phenotypic and genotypic methods briefly reviewed here is far from complete, our choice being to highlight only the most suitable techniques to differentiate among microorganisms of probiotic interest. While understanding the winning approach that can be obtained by the combination of different identification and classification procedures, the discriminatory role of molecular techniques is increasingly important even if only the best laboratories are able to provide results with these advanced methods.

Routine diagnostic laboratories continue to identify isolated strains by means of the classical methods, which represent cheap, readily available, and easy to handle options. However, there is a growing tendency for the identification procedures to become polyphasic, especially when the main target is to support a claimed effect of a probiotic microorganism.

The strain-specific nature of health effects claimed for different probiotic strains is suggested by several results mainly obtained *in vitro* on different strains (8–10), while few comparisons of strain specificity for health beneficial effects have been conducted in humans (76).

A well-known example of the important role of molecular techniques in the correct identification of probiotic strains is provided by the study of two commercial preparations of probiotics leading the authors to conclude: ‘… we found that two commercial preparations of probiotic bacteria purported to contain *B. subtilis* contain instead *Bacillus* species that are closely (Biosubtyl) and distantly (Enterogermina) related to *B. subtilis*’ (77).

Two years later, an Italian team verified the real taxonomic position and looked carefully at the phenotypic and genotypic features exhibited by the four *Bacillus* strains isolated from the spore mixtures of the product Enterogermina present on the market since 1975. Their polyphasic approach led authors to the finding that all of the strains ‘… belong to a unique genospecies, which is unequivocally identified as the alkali tolerant species *Bacillus clausii* ’ (78).

Thus, it is important that the identity of specific strains declared on the product label is the result of a carefully selected combination of suitable phenotypic and genotypic analytical methods.

Furthermore, the need for ‘protection’ of the producer or owner of specific strains is an additional aspect to be considered, to avoid the use of these strains in commercial products without the consent of the owner.

Only such a policy by probiotic producers could give the right emphasis to the significance of strain-specificity and thus provide health authorities with accurate tools to better evaluate the health benefits claimed by each probiotic-based product. Finally, a clear and unequivocal identification and classification of the microbial strains contained in these products can assume a pivotal role should they be suspected of being associated with the onset of a disease in a consumer.
